# Cancer research and the mainstream of biology

**DOI:** 10.3389/fcell.2025.1623849

**Published:** 2025-07-02

**Authors:** Toivo Maimets

**Affiliations:** Institute of Molecular and Cell Biology, University of Tartu, Tartu, Estonia

**Keywords:** cancer, oncogenes, somatic mutation theory, development, reductionism

## Abstract

John Cairns, a British molecular biologist, has pointed out that biology and cancer research have always developed together, and cancer theories have followed “whatever branch of biology happens at the time to be fashionable and exciting”. Indeed, following the long historical development of biological thought confirms this observation. However, tumour theories have never been merely a “fellow runner” to more modern biology theories. Cancer is an exceptionally large medical and economic problem, and the practical results of cancer research are carefully followed and critically analysed by the community. If the expected results do not arrive and the scientific data do not fit into the old theory, then the theory must be corrected. In other words, tumour theories not only derive from the prevailing biological worldview, but they also influence and, if necessary, actively change it. That is exactly what we are witnessing today–the ruling reductionist Somatic Mutations Theory (SMT) does not explain many new experimental findings and extensive research over the last 50 years has not brought major breakthroughs in cancer treatment. This century brings back the attention to developmental biology (embryology) in connection with the epigenetic revolution in biology, and the causes of tumours are searched for in the disorders of differentiation of cells/tissues and communication between them in the organism.

## Introduction

John Cairns, a British molecular biologist, has pointed out in his book “Cancer, Science, and Society” that biology and cancer research have always developed together. “Invariably, at each stage,” Cairns wrote more than 40 years ago, “the characteristics of cancer cell have been ascribed to some defect in whatever branch of biology happens at the time to be fashionable and exciting; today, it is molecular genetics” (Cairns, 1978).

Indeed, following the historical development of biological thought confirms this observation. The first known descriptions of tumours come from the so-called Edwin Smith’s papyrus, from about 3,000 years BC, where it is recognized that tumour is a deadly disease that has no cure. Hippocrates (ca 460-370 BC) derived the name “cancer” (καρκινος) and until the birth of cell theory, tumours were treated according to humoral theory and treated accordingly with diet, grafting and laxatives. In the XIX century, tumours were explained by cell biology and embryology, in the XX century initially by viral tumour theory, and later by mutations in the DNA (SMT, somatic mutation theory).

However, tumour theories have never been merely a “fellow runner” to more modern biology theories. As cancer is an exceptionally large medical and economic problem and as such very burdensome for society, cancer research outcomes are closely monitored. And if the expected results do not arrive and the ever-changing scientific data do not fit into the old theory, then the theory must inevitably be corrected. For example, Hippocrates claimed that an excess of one of the four body fluids (humors), black pile, is the cause of tumors, and this position was also carried over to Claudius Galen’s (130-200) medical teaching. However, regulating the balance of the four body fluids with diet, laxatives, or bloodletting did not produce significant results in terms of tumour treatment, and this called into question the validity of the theory of humors. Several other theories had the same fate (for example, Stahl-Hoffman and Hunter’s lymph theory in the 18th century or Zacutus Lusitani’s and Nicholas Tulp’s theory of cancer as an infectious disease in the 17th-18th centuries) (American Cancer Society 2024). In other words, tumour theories not only derive from the prevailing biological worldview, but they also influence and, if necessary, actively change it. That is exactly what we are witnessing today. This century brings back the attention to developmental biology (embryology) in connection with the epigenetic revolution in biology, and the causes of tumours are searched for in the disorders of differentiation of cells/tissues and communication between them.

## Genetics and embryology (developmental biology)

A large amount of literature on the biology of tumours begins with statements like “cancer is a genetic disease” or “cancer is a disease of genes.” According to this view, cancer begins with genetic changes - “somatic mutations” that create new tumour properties for cells. Eventually, thanks to such mutations, the cells acquire mobility, invasiveness, and the ability to create metastases. Hence, cancer is a genetic problem. This is a brief summary of the common theory of tumours - somatic mutations theory (SMT).

This has not always been the case. Such a view was formed only by the middle of the last century, together with Modern Synthesis (MS), reductionism in biology and the idea of “selfish genes” that use organisms as means to travel from generation to generation and describe the “construction plan” or “blueprint” of the whole organism. The genetic theory of tumours postulated genes that are activated by mutations and lead to the promotion of tumour formation (oncogenes) and others, whose inactivation contributes to tumour formation (tumour suppressor genes).

In the 19th century, the causes of tumours were seen elsewhere - the tumours came either from less differentiated cells that had been delayed in development (“embryonic remnants”) or from cells that had been dedifferentiated for some reason (Stanger and Wahl, 2024).

“Oncogenesis is blocked ontogenesis,” wrote Julius Cohnheim in 1875. Tumours are produced not from normal adult cells, but from “embryonic” remnants - cells that are “left behind” in their development in the adult body (“*embryonale anlage*” or “*verirrte Keime*”) (Cohnheim, 1875). The change in the surrounding environment causes otherwise silent embryonic cells to become malignantly proliferating. They do not differentiate normally but remain similar to embryonic tissue. In conclusion, tumours are not a genetic problem, but a problem of developmental biology.

Francesco Durante, a surgeon and pathologist, summarizes the picture of the emergence of tumours in 1874 as follows (Triolo, 1965; Stanger and Wahl, 2024):

Elements which have retained their … embryonal characteristics in the adult organism, or that have regained them through some chemico-physiologic deviation, represent … the generative elements of every tumor variety and specifically those of a malignant nature. Such elements may remain enclosed within matured tissues for many years, giving no indication of their presence, until an irritation—a simple stimulus suffices—rekindles their vital cellular activities.

Durante describes here two possible embryonic intermediate stages through which the tumour is formed. Firstly, it may be a cell that has stopped in the differentiation process before the end, and secondly, it may be a cell that has regained embryonic traits. As we will see below, both options are also under active discussion in 21st century biology.

At the same time, British developmental biologist John Beard pointed out the similarities between early embryonic development and tumour malignancy (Burleigh, 2008). Trophoblast cells, which are the first product of cell differentiation in the development of human embryo (produced in an embryo of about 4-5 days of age), are very similar to invasive tumours: these cells penetrate the uterine wall, multiply very actively, provide themselves with blood vessels, and suppress the immune system of the mother’s body so that fetal growth is possible. It is these properties that are also associated with metastatic processes in the body. Beard suggested that the aggressive tumours come from displaced trophoblast cells.

Modern studies also show the link between stem cells and tumour cells. Transplantation of normal stem cells from the genital wall into mouse testes results in malignant tumours - teratomas (Mintz and Illmensee, 1975). Teratomas contain cells from different developmental lines in different phases of differentiation. However, if these same cells of the genital wall were taken from genetically sterile mice (who do not have gamete stem cells), there will be no teratomas. Moreover, when teratoma cells are injected into normal blastocysts, teratogenesis is suppressed. These tests showed that a normal (germarium) stem cell can produce a tumour in an appropriate environment and, on the other hand, a normal environment is able to suppress tumour production processes (Stevens, 1964; Stevens, 1981). Such works confirm Durante’s hypothesis that tumours can be formed from stem cells if there is a suitable environment.

The idea that tumours arise due to changes (mutations) in cell chromatin came up in 1902 by the embryologist and zoologist Theodor Boveri (Boveri, 1914). This was direct consequence of Boveri-Sutton’s chromosome theory, according to which chromosomes are carriers of genetic material. Earlier, David von Hansemann had described aneuploidy of chromosomes in tumour cells (additional chromosomes, missing and faulty chromosomes) in 1890 and Boveri concluded that these effects were the cause of the tumours. Later, upon learning the structure of DNA, this position was clarified for the role of DNA mutations in tumour formation. Von Hansemann himself considered chromosome changes to be the result, rather than the cause of tumours rather (Seyfried et al., 2014). Boveri also postulated the presence of cell cycle control points (*Hemmungseinrichtungen*), tumour suppressor genes (*teilungshemmende Chromosomen*) and oncogenes (*teilungsfoerdernde Chromosomen*). He speculated that the tumours were caused by radiation, physical/chemical damage, or pathogenic microorganisms. The theory of chemical carcinogenesis was much older - already in 1775, Percival Pott observed that there are many scrotal tumours among chimney sweepers.

By the beginning of the XX century, it was clear that there are two equally important “meta-themes” in biology: how different characteristics of the organism are created during the course of individual development (this was a subject of embryology, later developmental biology), and how characteristics are passed on to subsequent generations (genetics). In 1910, Thomas Hunt Morgan rephrased the traditional contradiction between preformism and epigenesis, calling it a contradiction between “particulate theory of development” and “theory of physicochemical reaction”. He writes: “Whichever view we adopt will depend first upon which conception seems more likely to open up further lines of profitable investigation … ” (Morgan, 1910). Although Morgan had begun as an embryologist, he noted that genetic research on fruit flies allowed him to ask important questions about genetics, but there were no comparable methods for studying embryology at that time. Although it was clear from the very beginning that all heredity only materializes through the processes of individual development, they are inseparable and affect (and shape) each other.

In the 20th century, major changes took place in biology theory, and with the advent of Modern Synthesis, genetics became the fundamental - and meantime the exclusive–component of it. The central axis of biological thought was around DNA and, above all, its mutations. The starting point of biological theories was reductionism, genetic determinism, and the assumption that there is a linear causative bond between the genotype and the phenotype (“one gene - one protein - one trait”). This was reinforced by the experiments of Osvald Avery, Colin MacLeod and Maclyn McCarty in 1944, according to which it was the DNA molecule that allowed the transfer of *Streptococcus pneumoniae* virulence to non-virulent strains (regrettably, later this experiment led to the erroneous conclusion that all traits of organisms are only transferable by DNA molecules). The subsequent discovery of the DNA structure (Watson and Crick, 1953) and the genetic code (Nirenberg and Matthaei, 1961) fixed the main directions of biological thought for the decades to come. SMT became the central theory of cancer formation.

## Oncogene concept

Michel Morange has thoroughly described the main discoveries, which led to the so-called “oncogene revolution” in the 1980s (Table 1; Morange, 1997). According to this paradigm, tumours arise due to an increase in the expression or structural changes of a small number of genes - oncogenes. Such oncogenes are activated by a number of different mechanisms: direct mutation; increased transcriptional activity due to the entry (insertion) of retroviral sequences into the vicinity of the oncogene in the genome; gene transfer in the genome (translocation) places them under the influence of highly active promoters; the entry of the oncogene into the retroviral sequence, together with which its highly active expression occurs. Oncogenes are also activated by multiplication (amplification) of its DNA sequences. In summary, cancer occurs due to changes in various oncogenes and is therefore their cooperative effect. Oncogenes encode various proteins that are involved in the control of cell proliferation: growth factors that control cell division; receptor proteins of these factors; proteins of signal pathways that transmit extracellular signals to the cell nucleus; nuclear transcription factors responsible for the expression of genes in rapidly reproducing cells. Later, tumour suppressors or anti-oncogenes were added, which could be inactivated by various mechanisms, and such inactivation contributed to the formation of tumours. For example, the gene *p53* (and the corresponding protein) has been mutated in more than half of human tumours.

**TABLE 1:** The most important discoveries underlying the concept of oncogenes (*modif.* Morange, 1997).

Year	Discovery
1911	Detection of the first cancer-causing virus (P. Rous)
1961	Gene regulation models (F. Jacob and J. Monod)
1969	Provirus model (R.J. Huebner and G.J. Todaro)
1976	The first cellular oncogene was found to be homologous to retroviral oncogenes (D. Stehelin, H. Varmus, M. Bishop)
1979–1980	Intercellular transfer of the transformed phenotype with a single gene (G. Cooper, R. Weinberg)
1982	Transformation was shown to occur as a result of a single point mutation of the cellular oncogene (Ras) (R. Weinberg)
1983–1984	Oncogene products are cell growth factors or growth factor receptors
1986	Identification of the first anti-oncogenes (tumour suppressors)

The concept of oncogenes replaced the position that prevailed just before that time, according to which the tumours were of viral origin. Indeed, when President Richard Nixon asked the U.S. Congress to announce the National Cancer Act in 1971, it was subtitled “Virus Cancer Program.” The aim of the programme was neither more nor less than to eradicate the tumours in the next 10 years $1.5 billion was earmarked for this, the National Cancer Institute was established, and a number of the best scientists of that time joined the program. However, the task turned out to be much more complicated. 50 years later, President Biden (February 2022) announced the “Cancer Moonshot” program, which was designed to reduce cancer deaths twice over the next 25 years (Ledford, 2022).

However, progress is not negligible - while in 1971 only half of the cancer patients lived for at least 5 years, in 50 years it had increased to two-thirds. For a number of cancers - lung, prostate, stomach, etc. there is a significant reduction in age-adjusted tumour mortality. However, for example for pancreatic and liver tumours, the trend is rather the opposite (American Cancer Society, 2019).

**FIGURE 1:**
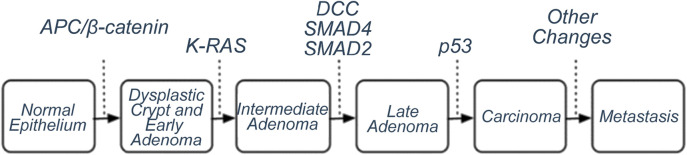
Canonical model of colorectal tumour formation by SMT (Fearon and Vogelstein, 1990). Genes whose mutations/activation lead from one stage to the next have been identified. Allegedly, 50%-85% of colorectal tumours behave according to this model.

By 1978, it was clear that the absolute majority of human tumours were not virus-related, and it was necessary to find a new bearing axis in cancer biology. The basis for the emerging concept of oncogenes was expressed already in 1974 by Howard Temin: mutations in cell genes lead to the development of tumours (Temin, 1974). This was accompanied by discoveries that the oncogenic transformation of cells and the formation of tumours were accompanied by many chromosomal rearrangements (see e.g. Cairns, 1981) and George Todaro clearly formulated the idea (although it had been suggested earlier) that the tumours were caused by abnormal production of molecules such as ligands of cell growth factor receptors in cells (e.g. Todaro and De Larco, 1978). The first oncogene was described in 1976 (*Src* found in birds’ Rous sarcoma viruses) and was immediately proposed as a regulator of cell growth and development (Stehelin et al., 1976). The idea that the formation of cancer is a multi-step process that occurs with mutations in some genes came from the fact that the probability of the occurrence of cancer increases exponentially over the years, and from the slope curve it was found that the number of relevant elementary events could be from 5 to 8. Bert Vogelstein developed a model of canonical colon tumour formation from normal intestinal epithelial cell to metastatic tumour, where changes in certain oncogenes and/or tumour suppressor genes could be demonstrated at each stage (Fearon and Vogelstein, 1990; Figure 1). True, it soon became clear that many colon tumours, however, do not follow this model, and as Robert Weinberg has expressed years later:

“…each tumor seemed to represent a unique experiment of nature, acquiring a unique set of mutant genes and in an unpredictable chronological order.” (Weinberg, 2014).

Central to the development of the oncogene concept were Robert Weinberg’s transfection experiments. The growth of normal cells (for example, in the Petri dish) is characterized by the so-called “contact inhibition” - the cells divide as long as they come into contact with neighbouring cells and then the division stops. As a result, a single-cell layer is formed on the dish. However, when different oncogenes were introduced into the cells, such contact inhibition disappeared and the “growth cups” developed - such cells were called (oncogenically) transformed. Weinberg’s work thus made it possible to find and characterize mutations that lead to the formation of active oncogenes and showed that the transformation of cells requires the modification of several genes (Land et al., 1983a; Land et al., 1983b; Ruley, 1983). True, there was constant debate about the extent to which such a transformation still reflects the actual oncogenesis process.

In 1982, Weinberg and colleagues found that it was enough to transfer a single mutated gene (from bladder carcinoma cells) to produce cell transformation. It was a gene called *Ras*, which had already been found in retroviruses. The real surprise, however, was that the tumour-causing *Ras* differed from normal by just one nucleotide. In 2014 Weinberg writes (Weinberg, 2014):

“For a brief moment in 1982, there was the illusion that cancer was as simple as it possibly could be—a normal cell differed from its neoplastic counterpart by one base out of three billion!”

However, he goes on to write:

“From the point of view of the reductionist hoping that a small number of molecular events might explain cancer, things went downhill from there for the next 30 years.

…

So, perhaps ironically, we have come full circle, beginning in a period when vast amounts of cancer research data yielded little insight into underlying mechanisms to a period (1980–2000) when a flurry of molecular and genetic research gave hope that cancer really could be understood through simple and logical reductionist thinking, and finally to our current dilemma. Once again, we cannot really assimilate and interpret most of the data that we accumulate.”

## The role of mutations in the formation of tumours - “drivers” and “passengers”

After it became clear that mutations play a role in tumour production processes, and in parallel with rapid developments in nucleic acid sequencing technologies (determination of nucleotide sequences of DNA and RNA), great attention was paid to the search for different mutations in very different tumours. A great deal of work was done here by the consortium The Cancer Genome Atlas (TCGA), led by the National Cancer Institute (NCI) and the National Institute of Human Genome (NHGI), which started in 2006. In total, tumour and corresponding normal tissue material was sequenced from 20 thousand patients and 33 different tumour types. A number of other tumour-related data were also collected.

It became increasingly clear that tumours do not arise due to mutations in a limited number of genes (5-8 as suggested earlier), but more and more potential oncogenes were added during sequencing. This made it difficult to understand, which of them were causally related to the development of cancer and which were not. It also became clear that mutations also accumulate in normal tissues as they age. They establish cell clones that may or may not become tumours (Wijewardhane et al., 2020). Now the question arose, which of these mutations are actually causally related to tumours, and how would we know that?

The mutations in the tumours were divided into two types - some are “drivers” (*driver mutations*) that cause tumours, while others are called “passengers” (*passenger mutations*) with no causative relationship to tumours. However, it turned out that such a division is not at all easy (Pradeu et al., 2023). What does causality mean? Some mutations may not directly cause the tumour phenotype, but may, for example, hinder the mitosis process and thereby increase the frequency of mutations in the cell. Are they supposed to be drivers? Therefore, the definition of drivers and passengers is not clear.

Functional data are used to distinguish between the two mutation types. For example, mutant genes are inserted into mice or cell lines. But the results obtained so far are often contradictory because the effects depend on the specific cell types and also on what other mutations exist in these cells - so the function of the gene depends on the genome and the cellular context.

Another way is to analyse many tumours of the same type, and if a mutation occurs in most of them, it could be concluded that it is a driver. The problem is that the molecular pattern of tumours in patients with the same diagnosis can be very different (Kumar et al., 2021) and different mutations are found in different parts of the same tumour mass. For example, different mutations were found in different areas of the same lung tumour, so it had to be stated that “… a biopsy taken from R3 might suggest treatment with an inhibitor of the … PI3K/mTOR signalling axis, and combination therapy. Conversely, a single biopsy from another region would suggest treatment with a BRAF inhibitor … ” (de Bruin et al., 2014).

The likelihood of any mutation in the tissue depends on many of the factors involved: the size of the gene, the position effect, the openness or fragility of the chromatin region, etc. Therefore, it is also difficult to define false positive and negative signals (Lawrence et al., 2013). Certain gene regions (hotspots) have been found that are more often mutable and thus give false positive responses (Hess et al., 2019) and deviations from the average rate of mutations in certain genome regions has been described (Monroe et al., 2022).

Pradeu, together with his co-authors, stresses that the concept of drivers/co-drivers itself is also changing and currently conflating two meanings (Pradeu et al., 2023). When originally “driver” signified a mutation that gave the cells a certain selective advantage or growth fitness (drivers drive clonal expansion (Maley et al., 2004)), it now includes mutations in any gene that plays some functional role in tumour (drivers drive the disease). These two approaches overlap only partially. For example, some mutations create a growth advantage, not a tumour process (Wijewardhane et al., 2020). Cell clones with mutations are also formed in non-tumour tissues during aging (Kakiuchi and Ogawa, 2021). Some mutations that give cells a growth advantage are enriched in tumour cells, while others are instead enriched in non-cancer tissues. This is the case, for example, with the genes *p53* and *NOTCH1* in the esophagus, where it is also thought that the *NOTCH1* mutations might in fact protect against tumours (Martincorena et al., 2018). Some mutations produce a tumour phenotype but are more neutral to the growth advantage or even inhibit cell growth, such as *SRSF2* mutations in haematopoietic cells (Bapat et al., 2018).

All in all, it should be noted that instead of some (5-8) oncogenes, hundreds of mutated genes have been found in tumours and it is very difficult to define which ones are causally related to the formation of tumours. It usually depends on the context - the effect of the activity of other genes, the environment and other cells in the tissue. The same mutation can be both driver, neutral and harmful. For example, mutations in the *KRAS* gene (Kirsten’s rat sarcoma) are the predominant driver of pancreatic duct tumours (PDAC), but a specific context is needed for cell transformation: chronic inflammation (Guerra et al., 2007) or specific acinar cells in which telomerase is activated (Neuhöfer et al., 2021).

Moreover, whether the mutation is a driver, neutral or damaging may depend on the heterogeneity within the tumour and the way the tumour has been previously treated (Swanton and Govindan, 2016). For example, in lung adenocarcinomas, the *EGFR* mutation T90M becomes a driver only if the tumour cells have been previously selected by treatment with EGFR inhibitors. Also, whether a particular mutation is the cause (driver) of the tumour or a related phenomenon (passenger) may depend on the cellular (tissue) context (Haigis et al., 2019).

Pradeu et al. observe:

“Overall, this shows that the concept of driver mutation is relative, not absolute, and it is important to disentangle its contribution either to clonal expansion and/or to oncogenic processes (partially overlapping processes)” (Pradeu et al., 2023).

There are even more problems with the connection of mutations and causation of tumours. There are tumours, where no mutations have found, such as teratocarcinomas and ependymomas (Martincorena and Campbell, 2015; Versteeg, 2014). Tumour cells with DNA mutations can be differentiated into completely normal developing cells, with the mutation remaining (McClellan et al., 2015). Also in normal tissues, mutations that are characteristic of cancer tissues and/or driver mutations (Martincorena and Campbell, 2015) are often found, with some mutations found in normal cells even giving protection against cancer (Zhu et al., 2019; Colom et al., 2021).

Cells have high phenotypic plasticity: one genotype can give very different phenotypes, therefore the classic Darwinian “mutation-selection” does not fully explain the development of tumours. Also, the mutations found may not have been selected, but instead have arisen from gene drift (Sottoriva et al., 2015).

There is an increasing accumulation of data according to which the behaviour of a gene as an oncogene or as a tumour suppressor gene depends on the context (see Monti et al., 2022 for many examples). For example, extracellular signals determine whether oncogenes direct skin cells to cancer or in the opposite direction (McNeal et al., 2021). The claudins can be both tumour-promoting and tumour suppressors, depending on the microenvironment (Li, 2021).

*c-myc*, one of the most canonical oncogenes, can actually cause differentiation and apoptosis in human embryonic cells (Sumi et al., 2007; Amati and Land, 1994). The activity of *c-myc* as an oncogene or suppressor for the production of tumours may depend, for example, on the density of cells (Lee et al., 1995) or the status of E2F1 signalling pathway (Cucina et al., 2006).

Monti et al. has also proposed a model of the various attractor states originally presented by Conrad Waddington in the phenotypic state-space landscape. Depending on internal and external limitations, different states of the Gene Regulatory Networks (GRNs) are created, which may give completely different effects from the point of view of tumour development (Monti et al., 2022).

De Magalhães recently found that of the 17,371 human genes encountered in at least one scientific article in the PubMed database, 15,233 (87.7%) are associated with at least one article on cancer. The number of genes mentioned in at least 100 articles was 4,186 and only three of them (*SLC26A5*, *PRPH2* and *CRYZ*) were not included in any cancer-related articles. So, he has put the title of his commentary: “Every gene can (and probably will) be associated with cancer” (de Magalhães, 2022). If almost any gene is associated with tumours in some way, then the concept of few “driver genes” does not seem to be relevant.

As has been said, tumour tissues usually contain a large number of DNA mutations, whereas mutations in cells of the same tissue can be different, and the spectrum of mutations also changes over time, starting from early stages to metastases. Up to 20,000 different point mutations have been found in breast tumours (Yost et al., 2012), in melanoma up to 333,000 (Berger et al., 2012). Already in 1920, David von Hansemann asked about the aneuploidies found in tumours, whether the mutations in tumours are the cause or consequence (Hardy et al., 2005).

Monti et al. (2022) summarize the role of mutations in cancer formation as follows:

“To sum up, we may confidently conclude that mutations are associated with tumors, even if they might be irrelevant as a primary cause (Boland and Ricciardiello, 1999), and hence ‘‘are not likely to play a dominant part in cancer’’ (Hua et al., 1997).

## Nucleus or cytoplasm

British geneticist Cyril Dean Darlington was one of the first to challenge the nuclear origin of tumours (Darlington, 1948). He was convinced that the causes of the tumours were derived from cytoplasmic elements - *plasmagenes*, which have later been considered to be, for example, mitochondria (Seyfried et al., 2014).

Several experiments with cell nucleus/cytoplasm transfer indicate the possible cytoplasmic nature of the causes of tumour formation. When the cytoplasm of normal cells is brought together with the nuclei of tumour cells, the tumour phenotype is suppressed (see Seyfried et al., 2014 for references). *In vivo* experiments have also shown that the tumorigenicity of many tumours decreased when their nucleus got into contact with the cytoplasm of normal cells - although tumour-associated mutations were still present in these nuclei. The transfer of tumour cells into the mouse embryo resulted in cells with a normal phenotype (which had still retained DNA mutations from tumours) (Li et al., 2003; Hochedlinger et al., 2004). Inverse experiments combining the cytoplasm of tumour cells and the nuclei of normal cells led to a tumorigenic phenotype (Israel et al., 1988; Petros et al., 2005). These results echo the views of C. D. Darlington.

## Cell differentiation, plasticity and tumours

All human cell types (about 250 in total) and different tissues are derived from a single cell - a fertilized egg. Embryonic development includes both quantitative changes (increase in the number of cells due to proliferation) and qualitative changes (different cell types are formed as a result of differentiation). Differentiated cells have a characteristic morphology and expression pattern of genes and proteins.

**FIGURE 2:**
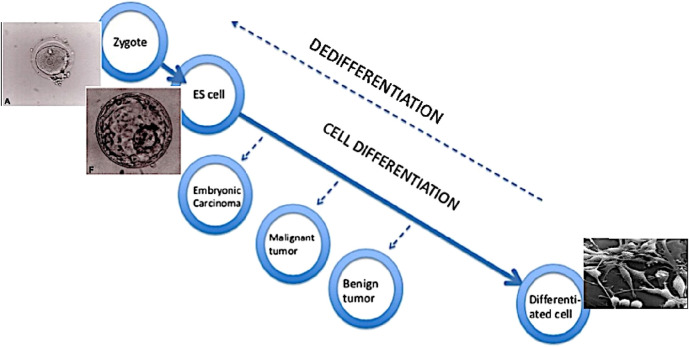
Loss and gain of cell developmental potential. According to concept of tumour as a problem of development, a tumour cell has an undifferentiated or low-differentiated status. In the course of differentiation from zygote to terminally differentiated cells, the process can stop and result in tumours - the less differentiated, the more aggressive. The same result can also be achieved through dedifferentiation of cells. ES cell - embryonic stem cell.

Cancer cells are characterised by a dedifferentiated or low-differentiated state. It has been known for decades that the less differentiated the tumour cells, the more aggressive the tumour is. There are two ways to develop a low-differentiated phenotype: the dedifferentiation of already differentiated cells, or the failure to complete the initial differentiation (Figure 2). (See also Francesco Durante’s views in 1874, above). For example, adenomas (benign tumours) in the intestine are formed either from Lgr5+ stem cells which are located in crypts, or from the already differentiated cells of the villi.

Different mechanisms participate in the respective differentiation retardation or dedifferentiation processes (Schwitalla et al., 2013). The gradients of morphogens and their disorders, inflammatory processes, as well as mechanical stresses and the microenvironment of tumours (TME) may play a role.

There are several additional data on the link between differentiation status and cancer development. In acute promyelocytic leukaemia (APL), which has a chromosome translocation resulting in the fusion gene of the retinoic acid receptor alpha (*RARα*), retinoic acid produces differentiation of leukaemia cells and achieves excellent clinical results (Degos et al., 1995). In pancreatic tumours, damage to the *PTF1A* gene is an important stage of tumorigenesis - it is an important gene for the differentiation of pancreatic acinar cells (Krah et al., 2015). Gene *IDH* mutations produce cholangiocarcinomas (a biliary tumour) due to disruption of hepatocyte differentiation (Saha et al., 2014).

In 2008, we demonstrated a direct link between tumour suppressor gene activity and cell differentiation (Maimets et al., 2008). The protein product level of the gene *p53* is quite low in normal cells, because it binds to the protein Mdm2, which is the E3 ligase of the ubiqitin pathway, and therefore leads to the degradation of the protein p53 in the proteasome. Low-molecular compound nutlin binds to the structure of Mdm2 at the same site that binds to the p53 protein, so there is no degradation of p53 and the level of the latter in the cell increases. In embryonic human stem cells, such an increase in the level of protein p53 leads to rapid differentiation of the cells, which is associated with a stop in the cell cycle (Maimets et al., 2008). These results show the link between oncogenesis and differentiation and also support the perspective of tumour differentiation therapy.

Several studies have investigated the possibility of reversing cancer process through induction of cancer cell differentiation. Pioneering research showed that teratocarcinoma cells injected into normal blastocysts were transformed into normal embryonic cells, developing into normal tissues and organs (Mintz and Illmensee, 1975). Differentiation therapy has yielded significant results in the treatment of acute promyelocytic leukemia (APL), where the combination of all-trans retinoic acid (ATRA) and arsenic trioxide (ATO) achieved a treatment efficacy of over 95% (Lo-Coco et al., 2013). Although differentiation therapy has been shown to be effective in APL, the application of such therapy in solid tumors is more challenging (Shin and Cho, 2023).

The concept of cancer stem cells (CSC) also points to the problem of insufficient differentiation in tumours. CSC is a cell that usually forms a small part of the tumour, but has the ability to renew, differentiate and create tumour tissue, being resistant to both chemotherapy and radiation therapy.

## Tissue organization field theory - TOFT

Following Kuhn (1962), the accumulation of anomalies in SMT, such as non-mutational tumors and context-dependent oncogene effects, signals a crisis necessitating a paradigm shift toward theories emphasizing tissue-level and developmental processes. Carlos Sonnenschein and Ana Soto counterbalance the theory of SMT with a theory that goes a step further in cell differentiation and communication disorders and assumes that cancer is a disease not of cells but of tissues. It is true that tissues are made up of cells, but at every biological level of organization (genes, cells, tissues, organs, organisms…) there are not only properties derived from the previous level, but also so-called emergent properties. Emergence refers to the appearance of overall behaviour in a complex system of many parts that cannot be predicted or understood by focusing just on what those parts themselves are like (Ball, 2023). Organisms include several levels of organisation, which differ from each other in the degree of constraints and openness. DNA is the most constrained and least open level of organisation (see Noble and Noble, 2023).

Sixty years ago, David Smithers warned against excessive reductionism stressing in many writings that cancer is a disorder of organisation of the human body, rather than a defect of cells (Smithers, 1962):

“Cancer is no more a disease of cells than a traffic jam is a disease of cars. A lifetime of study of the internal-combustion engine would not help anyone to understand our traffic problems. The causes of congestion can be many. A traffic jam is due to a failure of the normal relationship between driven cars and their environment and can occur whether they themselves are running normally or not.”

Regrettably, the next decades of cancer research went to an even lower level of organization, looking for causes and solutions in the DNA sequence. Philip Ball emphasizes in his recent book “How Life Works”:

“… the way (perhaps the only way) to get deterministic outcomes from noisy components is to rely on causal emergence: most of the causal phenomena, be it the state of cell or brain or the behavior of an organism, must arise at higher levels” (Ball, 2023).

A comparison of SMT and TOFT theories is shown in Table 2 (Sonnenschein and Soto, 2020). As the biggest difference between SMT and TOFT, the authors point out that if the centre of the SMT is the “independent cell,” then TOFT is the story of “development that went wrong.” It can also be seen from this sentence that it is actually an extension of the theory of “cancer as a developmental biological problem,” but its usefulness lies in the clear identification of several critical points, especially intercellular communication. In SMT, the “default” state of cells in an organism is arrested cell cycle that requires the action of oncogenes to initiate tumour processes, whereas in TOFT, the main state of cells is continuous proliferation and mobility, and only the surrounding cells keep it under control. Whereas these controlling cells can be very different tissue components (so-called morphogenetic field). So, the question now is not what triggers cell proliferation and mobility, but, on the contrary, what are the mechanisms inside the tissue that act as a brake on proliferation and mobility in the normal state and whose damage causes tumour formation.

**TABLE 2:** Comparison between the SMT and TOFT (Sonnenschein and Soto, 2020).

	SMT	TOFT
Premise 1: What is the default state of cells in multicellular organisms?	Proliferative quiescence. Cells require stimulation by external (“growth factors”) and/or intrinsic (“oncogenes”) factors	Proliferation with variation and motility. Regulation of constitutive cell proliferation and of motility is exclusively exerted by external and/or intrinsic inhibitors of these functions.
Premise 2: How does the process of carcinogenesis take place?	Changes in the DNA of the founder cell makes this cell unable to control its proliferation. As a consequence, a neoplasm will be formed.	Carcinogenesis is “development gone awry”. Chronic abnormal interactions between mesenchyme/stroma and parenchyma of a given morphogenetic field are responsive for the appearance of a neoplasm.
Level of biological organization at which carcinogenesis takes place	Cellular	Tissue
Target disrupted by the carcinogenic insult	DNA	Morphogenetic field
Role of DNA mutations	Causal	Irrelevant, epiphenomenon
Consequence of the insult	1. Uncontrolled cell proliferation. 2. Formation of a clonal neoplasm in which all cells are mutated in the same gene(s) affecting the control of cell proliferation. 3. Additional mutations are invoked by most researchers to explain metastasis.	1. Altered tissue structure involving hyperplasia, metaplasia, dysplasia, and carcinoma. 2. Formation of polyclonal neoplasms. 3. The constraints imposed by the tissue to its cells are impaired. As a result, cells express their default state (i.e. proliferate and migrate) thus causing tumour growth, invasion, and metastases.
Weaknesses and strengths of theories of carcinogenesis	1. Failure in explaining “foreign-body” carcinogenesis due to a lack of induced DNA mutations by physical or inert materials. 2. Failure in explaining the normalisation/”maturation” of cancer tissues when they undergo “spontaneous regression”. 3. In principle, no objection in explaining germline cancers by DNA mutations that may alter the control of cell proliferation.	1. Explains “foreign-body” carcinogenesis as an unspecific tissue disruption of a morphogenetic field. 2. “Spontaneous cancer regression” is compatible with the TOFT. Tissue recombinants show that cancer cells (even those carrying alleged “oncogenic” mutations) are “normalized” when placed in homotypic “normal” tissues. 3. The TOFT explains germline cancers through a morphogenetic field effect because the mutation is present in all cells in the affected organism.
Corollary	Irreversibility. “Once a cancer cell, always a cancer cell.”	Reversibility. Due to spontaneous and induced normalisation, cancer is not destiny.

## Coda

Now the question may arise, whether genes have anything to do with the development of tumours at all? Of course they do, but like all other biological processes, genes are just a resource that cells use to achieve one or another result. This is a complex resource because DNA sequences are far from unambiguous (in the terms of “one gene - one protein - one phenotypic trait”). A single gene produces a wide variety of products (RNA and protein molecules) that are used by the cell in different combinations. And gene networks work in a redundant way - if for some reason one genetic pathway does not work, it is often possible to achieve the same goal by using other gene combinations. The causes of tumours cannot be reduced to the level of DNA alone, as they are a disease of cells and tissues that contain new emergent causes, which do not appear at lower levels of biological organization. It is now time to think, how the cells get access to genetic information they contain according to their emergent needs? What are the actions of the cells/tissues - or even whole organisms - to selectively use the potential of their DNA?

Biological science has always depended on existing methods and models. If there was no microscope, no questions could be asked about the cells and the microscopic world. In the biology of tumours, there is a clear need today for new methods to ask questions at higher levels of biological organization than DNA and cells. The organoid method is one of the examples - three-dimensional cell cultures that can be cultivated *in vitro*. They simulate tissue properties (structure, function, interactions and spatial positioning with other cells and extracellular matrices) and have been increasingly used in research on various diseases in recent years (Fang et al., 2023). Biobanks of organoids, models of co-cultivation of organoids and immune cells, and organoids-on-chip methods will bring new developments to the study of tumour biology in the near future. There will certainly be new opportunities for combining organoid technology with real-time imaging (real-time imaging technology) and three-dimensional bioprinting techniques (3D bioprinting technology). Such new approaches will allow to reduce the restrictions arising from long-running reductionism, both on the theory of tumours and on practical solutions.

Considering the current trends in biology, two directions can be foreseen in which cancer research will develop more and more actively in the future. First, with new methods and ideas in developmental biology, the concept of tumour as a disease of development is becoming very important, and so does the study of epigenetics. At the same time, attention should be drawn to the fact that the term “epigenetics” is used in two senses. For molecular biologists, epigenetics is “a process that affects the expression of specific genes and is passed on to daughter cells, but not involve changes in DNA sequence” (Lodish et al., 2016). However, Conrad Hal Waddigton (1905-1975) originally defined epigenetics as “the branch of biology that studies those causal interactions between genes and their products that create a phenotype”. Waddington’s epigenetics describes the divergence between an organism’s genotype and phenotype that occurs during development (Waddington, 1942). David Haig has pointed out that for Waddington, epigenesis was similar to the discipline that we today call the biology of individual development (Haig, 2004), and it is this type of epigenetics that seems to offer new ideas and solutions in further cancer research.

It also becomes important to direct attention to higher levels of the organism rather than to the cell (or gene). After all, tumour formation is not directly destructive to the tumour cell itself. On the contrary, the tumour cell becomes immortal and can exist indefinitely under suitable conditions - like a cervical tumour cell line HeLa, which has been propagated in laboratories around the world since 1951. As a result of a tumour, the whole organism dies (and then, of course, its cells as well), therefore the tumour must be seen as a disease of higher level of organization. Complex wholes are inherently more than the sum of their parts, because the properties of each individual part depend on the context of that part within the whole in which it operates. Denis Walsh has reminded that since the Modern Synthesis our understanding of evolution has focused only on the genes and populations. The organism has been completely overshadowed in the landscape of evolutionary thinking. This is because MS treats organisms only as objects and not as agents. Organisms, Walsh asserts, require a special theory, an “agent theory,” as opposed to the “object theory” currently prevalent (Walsh, 2015; Walsh, 2018).

However, if we start from cancer as an organizational disease, then all its levels become important, from genes to the econiche, and no level is privileged (Noble and Noble, 2023). If a complex system has properties or functions that its individual parts do not have, then these are emergent properties that appear only when the individual parts relate to the whole. It is also clear from here, why only studies at the DNA (or cellular) level are not sufficient to understand tumours.

In this context of organicism it is appropriate to remind (and agree with) Smithers (1962) and Ball (2023) (cited above). Cancer is a disorder of organisation of the human body, rather than a defect of cells.

Cancer research is constantly developing along the mainstream of biology, while at the same time changing it. As new experimental technologies emerge and new data accumulate, it can revitalise old ideas and give them new meanings.
